# Biometrics of complete human pregnancy recorded by wearable devices

**DOI:** 10.1038/s41746-024-01183-9

**Published:** 2024-08-12

**Authors:** Lauryn Keeler Bruce, Dalila González, Subhasis Dasgupta, Benjamin L. Smarr

**Affiliations:** 1 UC San Diego Health Department of Biomedical Informatics, University of California San Diego, San Diego, CA USA; 2 Bioinformatics and Systems Biology, University of California San Diego, San Diego, CA USA; 3 Shu Chien-Gene Lay Department of Bioengineering, University of California San Diego, San Diego, CA USA; 4 San Diego Supercomputer Center, University of California San Diego, San Diego, CA USA; 5 Halıcıoğlu Data Science Institute, University of California San Diego, San Diego, CA USA

**Keywords:** Reproductive biology, Physiology

## Abstract

In the United States, normal-risk pregnancies are monitored with the recommended average of 14 prenatal visits. Check-ins every few weeks are the standard of care. This low time resolution and reliance on subjective feedback instead of direct physiological measurement, could be augmented by remote monitoring. To date, continuous physiological measurements have not been characterized across all of pregnancy, so there is little basis of comparison to support the development of the specific monitoring capabilities. Wearables have been shown to enable the detection and prediction of acute illness, often faster than subjective symptom reporting. Wearables have also been used for years to monitor chronic conditions, such as continuous glucose monitors. Here we perform a retrospective analysis on multimodal wearable device data (Oura Ring) generated across pregnancy within 120 individuals. These data reveal clear trajectories of pregnancy from cycling to conception through postpartum recovery. We assessed individuals in whom pregnancy did not progress past the first trimester, and found associated deviations, corroborating that continuous monitoring adds new information that could support decision-making even in the early stages of pregnancy. By contrast, we did not find significant deviations between full-term pregnancies of people younger than 35 and of people with “advanced maternal age”, suggesting that analysis of continuous data within individuals can augment risk assessment beyond standard population comparisons. Our findings demonstrate that low-cost, high-resolution monitoring at all stages of pregnancy in real-world settings is feasible and that many studies into specific demographics, risks, etc., could be carried out using this newer technology.

## Introduction

Pregnancy remains surprisingly underexplored at high temporal resolution, despite the importance to every single human, and the emergence of new tools in the form of wearable sensor devices (“wearables”) that allow efficient longitudinal measurement of physiology. In the United States, normal-risk pregnancies are monitored with the recommended average of 14 prenatal visits, beginning with monthly appointments from 8 to 28 weeks, biweekly until 34 weeks, and weekly check-ins until birth^1^. This infrequent scheduling has thus only enabled a low-resolution understanding of a few biometric modalities throughout pregnancy^2–4^. Wearables might be used to improve the continuity of pregnancy monitoring, but to date no wearable-derived high temporal-resolutions descriptions have been published examining this potential value across all stages of pregnancy.

Wearables have emerged as a tool for describing, monitoring, and even predicting illnesses, both acute (COVID^5–7^, flu^8,9^) and chronic (Diabetes^10,11^, Atrial fibrillation^12,13^). Continuous monitoring during pregnancy is now possible and could lead to similar functionalities for pregnancy – high-resolution description, surveilling of trajectories, and prediction of events (adverse complications or positive events, as in labor prediction)^14–19^. Decision support tools using these data would provide an opportunity for early detection of pregnancy abnormalities and reduction of maternal and infant morbidity and mortality^20^.

Many recently published studies have focused on assessing accuracy, feasibility, and acceptability of wearable devices with regards to pregnancy^20–25^, developing models or protocols for implementing real-world continuous monitoring of pregnancy^22,26,27^, monitoring or diagnosing specific conditions such as gestational diabetes^28–30^ or designing interventions to implement lifestyle changes during pregnancy^31–33^. Most studies that have focused on analyzing patterns of change have explored continuous activity, heart rate (HR), heart rate variability (HRV), or sleep throughout pregnancy, but did not include the data before or at conception and generally included low numbers of participants^6,18,32–35^. The two studies that included data prior to conception analyzed HR and HRV^36^ and distal body temperature (DBT)^37^ using two different wearables, included 18 and 30 participants respectively, and have set groundwork for the work presented here.

We have previously shown that pregnancy onset can be clearly identified through changes in continuous temperature captured by a wearable^37^. We hypothesize that other modalities will show different patterns of change, in as far as they reflect changes in different physiological systems. Many pregnancies are suspected to experience naturally arising terminal complications in the first several weeks—often before the pregnant person is aware of the pregnancy^38–40^. We, therefore, hypothesize that these patterns of change over time should look different when reflecting different outcomes, as in pregnancies that come to term versus those that experience early fetal loss (EFL).

In this manuscript we attempt to provide high-resolution trajectories of pregnancy across time, from before conception through to after delivery, using multiple measurement modalities, generated on the same individuals from the same device throughout. By providing these unique views into the pregnant physiology from 97 full-term pregnancies and 23 instances of early fetal loss (EFLs), we aim to test two hypotheses: (1) that high-resolution data across pregnancy provides insight into variance across time, across individuals, and between the modalities used; (2) that such data provide information different than and so complementary to current risk categories.

Through retrospective analysis of continuous, multimodal physiological records from 97 full-term pregnancies and 23 pregnancies that experience EFL, we confirm that multiple physiological modalities captured from off-the-shelf wearable devices allow for the construction of high-resolution full-term pregnancy trajectories. We find that different sensor modalities reflect different changes at different times across pregnancy, which is important to inform future device engineering and experiment design choices targeting specific times of pregnancy or specific changes. These full-term profiles further allow us to identify early deviations in high-risk individuals, as well as in pregnancies that experience EFL, demonstrating that continuous, real-world monitoring across all of pregnancy is feasible and yields distinct insights. Recent reviews have pointed out the need for high-resolution physiological time series analyses across pregnancy^16^. Here, we provide such profiles from before conception to several months post-delivery, along with analyses that confirm potential uses of these profiles in augmenting risk assessments using such data.

## Results

**Fig. 1:**
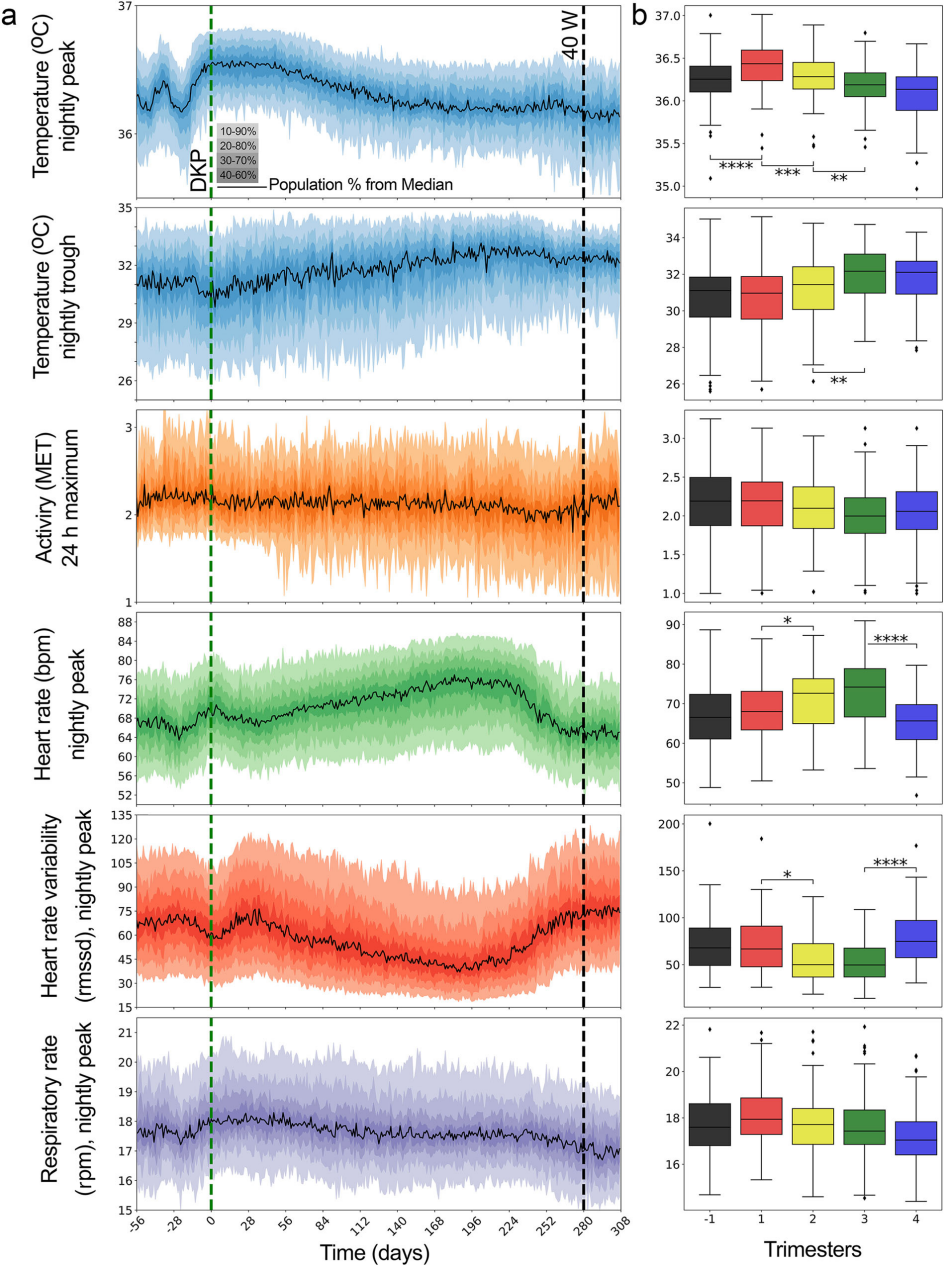
Multimodal trajectories reveal distinct changes across trimesters. **a** Median value (black solid) and quantiles (color) shaded for nightly peak temperature (blue), nightly trough temperature (blue), 24-h peak activity (orange), nightly peak Heart Rate (HR; green), nightly peak Heart Rate Variability (HRV; red), nightly peak Respiratory Rate (RR ; purple). All pregnancies were aligned by ‘date know pregnant’ (DKP, green dashed line) and ended within 3 weeks of the 40-week line (40 W, black dashed). **b** Pregnancy profiles displayed as boxplots (box = interquartile range (IQR) 1^st^ to 3^rd^ quantile, median = center line, lower whiskers = 1.5 * IQR below the 1^st^ quantile, upper whiskers = 1.5 * IQR above the 3^rd^ quantile) for 97 full-term pregnancies by trimester (trimester: -1 = -13 to 0 weeks prior to onset, 1 = 0 - 12 weeks of pregnancy, 2 = 13 - 26 weeks of pregnancy, 3 = 27 to 40 weeks of pregnancy, 4 = 40 to 53 weeks) by data modality. Bonferroni corrected *p*-value annotations for 4 comparisons: * : 2.5e-3 < *p* <= 0.0125, **: 2.5e-4 < *p*, ***: 2.5e-5 < *p*, ****: *p* <= 2.5e-6.

For the 97 full-term pregnancies, the 10th, 50th, and 90th quantiles by trimester are displayed in Table 1 and visualized in Fig. 1a. Nightly peak temperature (°C) and nightly trough temperature revealed two separate trends. Nightly peak temperature (Fig. 1b, first row, Table 2) was significantly different between the time prior to pregnancy (-1 trimester) to the first trimester, first to second trimester, and second to third trimester. The profile of the nightly peak temperature (Fig. 1a, first row) showed the pre-pregnancy ovulatory cycle between -60 and -30 days prior to the date of known pregnancy. Following conception, instead of adhering to the expected temperature decrease seen after the luteal phase during menstruation^41^, temperature increased past the pre-pregnancy temperature peak and slowly decreased across the pregnancy to lower than the pre-pregnancy average temperature. Nightly trough temperature was only significantly different (Table 2) between the second and third trimester (Fig. 1b, second row) and the overall trend was opposite to peak temperature, with a decrease in nightly temperature trough at pregnancy onset followed by a steady increase until the day of delivery (Fig. 1a, second row).

**Table 1:** Participant summary statistics by trimester

Data Type		Trimester				
		-1 (91 days)	1 (91 days)	2 (91 days)	3 (97 days)	4 (91 days)
Nightly Peak Temperature	μ(# days)	57	88	86	92	75
	quantile					
	10	35.96	36.13	36	35.88	35.72
	50	36.27	36.44	36.28	36.18	36.14
	90	36.52	36.71	36.56	36.45	36.43
Nightly Trough Temperature	10	28.22	28.23	29.33	29.94	30.28
	50	31	30.97	31.43	32.28	32.08
	90	32.85	32.74	33.24	33.7	33.25
24 h Peak Activity	μ(# days)	19	48	59	69	62
	quantile					
	10	1.8	1.75	1.61	1.66	1.62
	50	2.2	2.19	2.1	2	2.03
	90	2.79	2.61	2.62	2.54	2.48
Nightly Peak HR	μ(# days)	53	83	84	90	74
	quantile					
	10	56.77	59.22	60.16	62.22	55.63
	50	66.91	68.01	72.63	75.25	66.06
	90	75.94	77.55	80.81	83.02	74.67
Nightly Peak HRV	10	34.51	34.66	27.49	24.29	40.46
	50	68.66	66.73	50.01	45.62	73.29
	90	106.53	105.89	97.1	90.26	112.96
Nightly Peak RR	μ(# days)	22	56	71	85	73
	quantile					
	10	16.15	16.35	16.08	16.07	15.7
	50	17.61	17.94	17.71	17.46	17.08
	90	19.87	20.12	19.9	19.62	18.82

Quantile values bounding the 10–90% quantile shading in Fig. 1a for the six data modalities for all full-term pregnancies by trimester. Average days (μ) by data modality for all 97 pregnancies (nightly peak/trough temperature as well as and Heart Rate/Heart Rate Variability produced the same average number of days with measurements respectively).

**Table 2:** Statistical comparisons of modalities by trimester

Data Type	Mann-Whitney-Wilcoxon Test U stat (*p-value*)			
	-1 vs 1	1 vs 2	2 vs 3	3 vs 4
Nightly Peak Temperature	2893 (<0.001)****	6215 (<0.001)***	5937 (<0.01)**	5265 (0.0647)
Nightly Trough Temperature	4794 (-0.82)	3814 (-0.023)	3488 (<0.01)**	4862 (0.43)
24 h Peak Activity	1792 (0.3)	2910 (0.54)	4058 (0.16)	3836 (0.82)
Nightly Peak HR	3856 (0.25)	3450 (<0.01)*	3839 (0.03)	6914 (<0.001)****
Nightly Peak HRV	4429 (0.68)	5593 (<0.01)*	5280 (0.11)	2206 (<0.001)****
Nightly Peak RR	1441 (0.21)	3132 (0.19)	3832 (0.49)	4772 (0.02)

Mann-Whitney-Wilcoxon test two-sided U-statistic and *p*-value for trimester comparisons in Fig. 1b. *P*-value significance thresholds with Bonferroni correction for 4 comparisons: * : 2.5e-3 < *p* <= 0.0125, **: 2.5e-4 < *p*, ***: 2.5e-5 <*p*, ****: *p* <= 2.5e-6.

### Differences by modality from pre-conception through post-delivery

The same analyses were performed for four other data modalities: activity (metabolic equivalent task/metabolic equivalents; MET), heart rate (HR; beats/minute, bpm), heart rate variability (HRV; root mean square of successive differences (rmssd)), and respiratory rate (RR; respirations/minute (rpm)) (Table 1). Median activity was not significantly different between successive trimesters (Fig. 1b, third row) though a slight downward trend was present as pregnancy progressed (Fig. 1a, third row, n.s.). HR was significantly different between the first and second trimesters and between the third and fourth trimesters (Fig. 1b, fourth row). In general, HR increased from a pre-pregnancy state to a local peak about 14 days following conception then briefly dropped, subsequently, trended upward until a few weeks before delivery (Fig. 1a, fourth row). HRV was also significantly different between the first and second trimesters and the third and fourth trimesters (Fig. 1b, fifth row) and mirrored the HR trend decreasing following conception and then decreasing until a few weeks before the day of delivery (Fig. 1a, fifth row). RR was not significantly different between any of the trimesters and displayed a slight decrease across pregnancies (Fig. 1a, b, sixth row, n.s.).

**Fig. 2:**
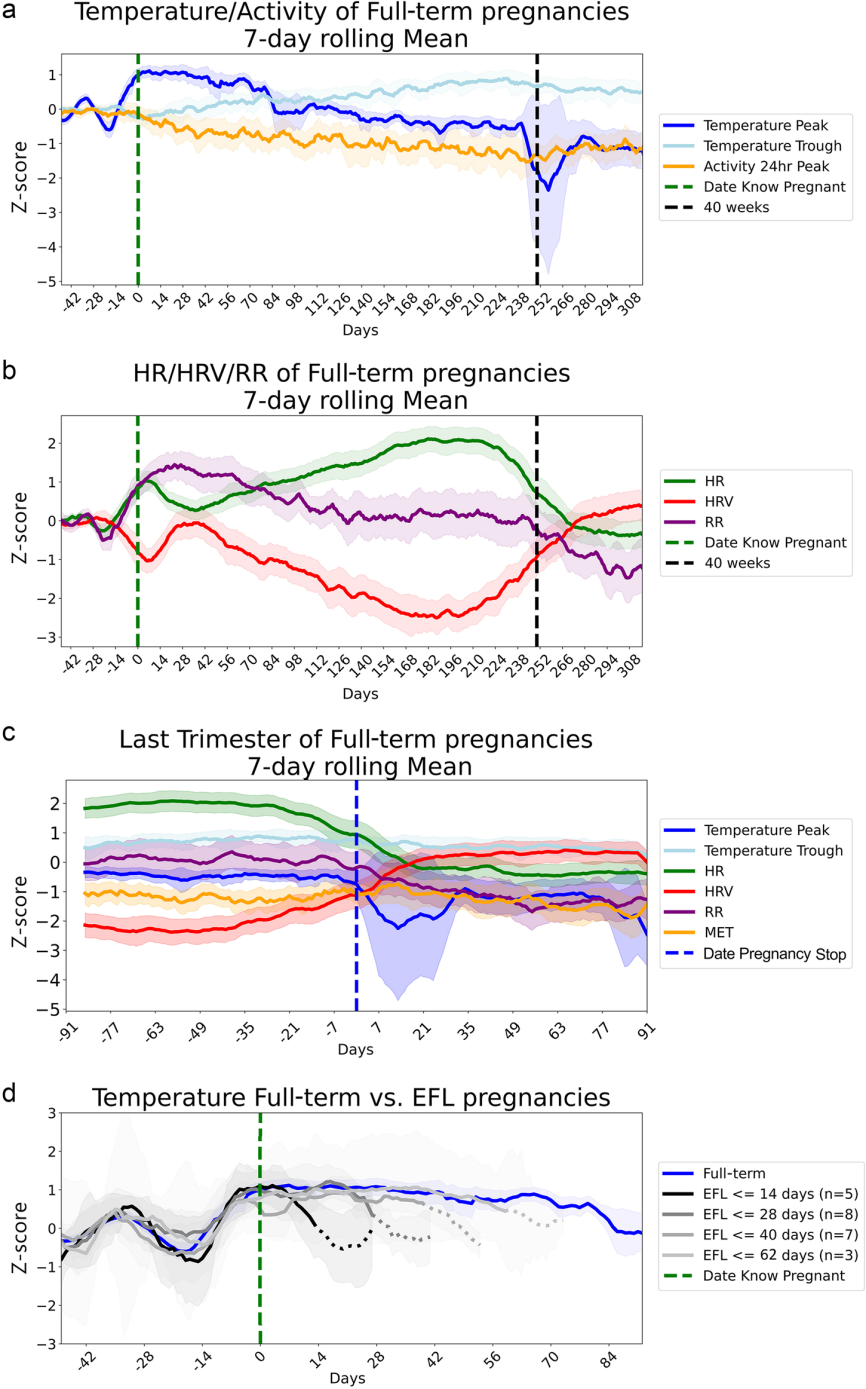
Normalized trajectories reveal signatures around conception, delivery, and early fetal loss. The Z-scored median (line) and 95% confidence intervals (shading) from the full-term population aligned by ‘date know pregnant’ for modalities derived from the wearable’s **a** thermistor (temperature nightly peak and trough) and accelerometer (24 h max metabolic equivalents (MET)) and **b** photoplethysmography (ppg; Heart Rate (HR), Heart Rate Variability (HRV), Respiratory Rate (RR)) sensors. **c** Z-scored values from all modalities aligned by ‘date pregnancy stop’ (DPS). **d** Z-scored temperature of Early Fetal Loss (EFL) pregnancies, grouped by gestation length past ‘date know pregnant’ (DKP) (darkest earliest: lightest latest) and full-term pregnancies (blue) aligned by DKP (green dashed line). Solid lines transition to dashed indicate all pregnancies in the group have ended.

Z-score transformation of the data against pre-pregnancy baseline values allows for the investigation of intra-individual deviation from a pre-pregnancy state. Population median and 95% confidence interval across the whole population reveal substantial relative change between modalities across pregnancy (Fig. 2a, b), consistent with the idea that different modalities contain complementary information. Aligning the data by ‘date pregnancy stop’ (DPS) shows patterns of each modality just before and following the day of delivery (Fig. 2c) revealed that all modalities also showed substantial changes following delivery, with several showing trends of change in anticipation of the end of pregnancy (Fig. 2c), with (for example) HR and HRV inverting relative to each other, returning to pre-pregnancy levels for the first time since conception.

### Observation of early fetal loss (EFL)

DBT of individuals whose pregnancies stopped within the first trimester, here referred to as early fetal loss (EFL) pregnancies, showed a significant deviation from the trajectory of full-term pregnancies around the time of EFL. When aligned by ‘date know pregnant’ (DKP, Fig. 2d), the temperature of individuals who go on to full-term pregnancies plateaued for the first 8 weeks of pregnancy then slowly decreased; by contrast, we found that EFLs occurred throughout the first trimester, and that EFL events at different times within pregnancy nevertheless shared a characteristic decline in nightly peak temperature around the time of EFL. We observed differences in the slopes of the nightly peak temperatures following an EFL event (Fig. 2d) and thus analyzed how time and EFL or full-term status affected temperature using a Generalized Estimating Equation (GEE). The data used for the GEE analysis included the 7 days post DPS from each EFL pregnancy (22 total) and 5 time-matched full-term pregnancies for each EFL (110 full-term pregnancies which were time-matched such that data from a full-term matched pregnancy started the same day as the EFL’s DPS to 6 days after the EFL’s DPS - note that DPS was a retrospective, recalled date, and so biomedical tests or confirmations at the time might potentially have come before or after the post-facto defined DPS). The GEE revealed a significant effect between day, category (EFL or full-term), and the day at which the EFL occurred (pregnancy length threshold, 3-way interaction, *p* = <0.01, Table 3). This is consistent with the apparent decrease in slope and further supports the notion that the physiological change associated with the EFLs is itself changing as a function of days from conception.

**Table 3:** Statistics of multimodal model of early fetal loss

Statistic	coefficient	*p* (z)	Confidence Interval [0.25, 0.975]	standard error
Intercept	1.11	<0.001 (3.4)	0.48, 1.76	0.33
Day	-0.23	<0.001 (-4.4)	-0.33, -0.13	0.06
Category	0.5	0.23 (1.2)	-0.31, 1.32	0.42
Day:category	0.25	<0.001 (4.4)	0.14, 0.36	0.06
Length threshold	-0.01	0.44 (-0.8)	-0.02, 0.01	0.01
Day: length threshold	<0.01	0.01 (2.5)	0, 0.01	<0.01
Category: length threshold	-0.01	0.22 (-1.22)	-0.03, 0.01	0.01
Day:category: length threshold	<-0.01	<0.01 (-2.7)	-0.01, 0	<0.01

Statistics from the Generalized Estimating Equations executed on peak distal body temperature data comparing Early Fetal Loss (EFL) pregnancies to time-matched full-term pregnancies in the 7-days following ‘date pregnancy stop’ (DKP) grouped by the pregnancy length threshold (<14, <28, <40, <63 days).

### Comparison of physiological signatures from broad risk categories

**Fig. 3:**
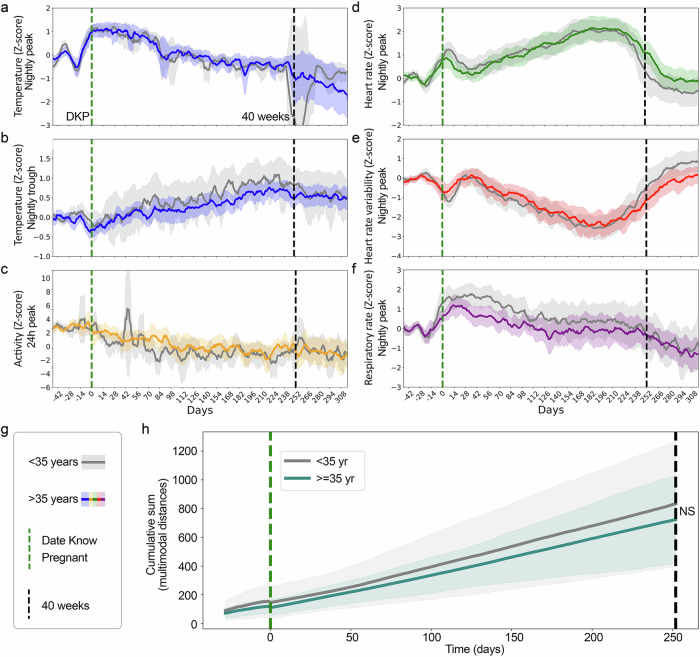
Trajectories in advanced maternal age are not on average different from other pregnancies. Median and 95% CI for individuals split by an age threshold of 35 years-old, with < 35 years of age represented in gray and >= 35-years of age in the respective data type color: **a** Nightly peak temperature (blue), **b** nightly trough temperature (blue), **c** 24 h peak activity (orange), **d** nightly peak Heart Rate (green), **e** nightly Heart Rate Variability (red), **f** nightly Respiratory Rate (purple). **g** Legend. **h** Cumulative multimodal (temperature peak and trough, HR, and HRV) distance from -4 to 40 weeks of pregnancy split by age (NS: no significant effect, Kruskal-Wallis U = 2.36, *p* = 0.12).

Increased age of pregnant people has been shown to correlate with higher-risk pregnancies, with the age of 35 commonly used as a threshold for increased risk^42,43^. However, the majority of pregnancies in people 35 or older are still healthy pregnancies. This raises the question of whether the risk category correlates to significant physiological differences between individuals in these categories absent pregnancy complications. We split the full-term cohort by the age threshold of 35 years (49 individuals < 35 years old, 48 individuals > 35 years old), to evaluate if there are differences between median values of the different modalities (Fig. 3). No statistically significant differences were found at weekly or 4-week aggregated resolutions between the less than and greater than 35-year-old groups.

Each modality alone might show too small a change to yield a significant difference, and yet in sum, the differences across modalities might reveal an overall physiological separation between the two categories as small differences accumulate across time. To test this possibility, we compared cumulative multimodal distance from 4 weeks prior to pregnancy to 40 weeks for individuals in each age group, and assessed whether small differences in each modality might add up to a larger net distance in a composite multimodal space. The final values in each age category were compared using the Kruskal-Wallis test, which found that no significant difference had accumulated by 250 days (U-stat = 2.36, *p*-value = 0.12).

## Discussion

Here we show high-resolution, multimodal physiological trajectories across the whole of human pregnancy, from cyclicity through conception and following delivery, from 97 full-term pregnancies. We found many significant differences across trimesters. Notably, different modalities had significant differences at distinct times across full-term pregnancies, suggesting that different physiological modalities may be more desirable for tracking pregnancy health at different times during pregnancy; each modality seems to likely be reflecting unique physiological processes, rather than being redundant. We then compared these trajectories to pregnancies for which the pregnant people reported early fetal loss and confirmed that the losses were associated with detectable changes in physiology throughout the first trimester: downward deflections of the temperature trajectory. The amplitude of deflection decreased with time from conception until it became non-significant toward the end of the first trimester. This loss of significance over time is likely an artifact of our relatively low N for late first-trimester Early Fetal Losses (EFLs); larger prospective studies with clinical verification of time of loss would clarify the physiological trajectories best suited for the detection or prediction of EFL across pregnancy, and might allow for the development of early alert or prediction systems. This confirms that continuous, longitudinal data can reveal previously unseen signs of clear relevance for pregnancy care decisions.

Complementing this existence proof, we rejected the hypothesis that current risk categories based on demographics necessarily correlate strongly with physiological change: we found that a broad risk category, pregnancy at an advanced maternal age, did not show significant differences from reference trajectories in any modality. This is probably not so surprising, as no one should expect that a person’s physiological radically changes on their 35th birthday, but we feel it is important to have explicit numerical demonstrations that longitudinal physiological measurements contain information substantially different—and hopefully additive to—current standards of risk assessment.

Together these two findings—observation of individual EFLs but not of differences between full-term pregnancies from broad risk category—support the hypothesis that individualized monitoring could augment broader risk assessments. Our work complements recent work showing that similar trajectories can be used to detect conception^41^ and also likely complications closer to delivery^18,19^. Here we show that this approach reveals a change in physiology consistent with a transition back to a pre-pregnancy physiological state following an EFL. Our findings do not support any one modality as ideal for revealing all of pregnancy, as each showed changes at different stages of pregnancy. Similarly, our findings suggest that algorithms for detection and prediction of adverse events should assess the changes to the patterns being detected across stages of pregnancy, as was observed for EFLs across the first trimester.

This retrospective analysis has several limitations that highlight important areas of future exploration before descriptions like those we have provided here should be trusted generally across diverse populations. First, all participants were current owners of the wearable device which may bias the data towards a more healthy and compliant population. Similarly, these individuals were likely of higher socioeconomic status (SES) and also educational achievement to have disposable income to spend on wearables, and data literacy to make use of the resulting outputs; they likely also do not come from disenfranchised groups, as by participating they appear to assume their experience should be counted. All of these conditions (higher SES, educational attainment, enfranchisement) are not evenly or well distributed across the population of people who might benefit from the potential improvements these technologies might make possible (i.e. everyone). Clearly more studies like this one, targeting specific (different) populations, and different conditions or outcomes of pregnancy, are necessary before any one finding or algorithm could be assumed to represent all pregnancy experiences accurately.

Additionally, reaching out to the community to request retrospective data allowed us to quickly collate pregnancies without restriction to specific recruitment locations; online recruitment also led to the inclusion of reports from many participants for which either physiological or self-report data were missing. In the interest of reducing participant burden and encouraging recruitment, surveys were of limited scope. Data were aligned based on the date each individual knew they were pregnant, presumably from a positive test, but this might be improved with reports of the start date of the most recent menstrual cycle, or with clinical confirmation in future prospective studies. Similarly, the lack of ethnicity and other non-age demographics (such as chorionicity or whether the pregnancy was medically assisted) and outcomes limit the number of subgroup comparisons possible in this data set. EFLs occurred in a comparatively small portion of participants; this allowed us to confirm that such events can be detected by wearables. We do not know from these data the cause or even exact timing of events that lead to the end of each pregnancy. That we found significant patterns associated with EFLs here suggests that sufficiently focused, prospective studies might be able to develop early detection or even prediction algorithms for the purposes of improving care around EFL events, as well as differentiating potential causes of spontaneous fetal loss. Because the end of pregnancy date was reported retrospectively by the participant, and not by a clinical definition or measurement, we do not attempt to interpret details around these events further here. Future studies could focus on high-risk pregnancies alongside normal-risk pregnancies, ensuring a diverse cohort of individuals (racially, ethnically, income status, education, etc.), and expanding the monitoring periods both before and after pregnancy.

Collecting more data with patient consent from electronic health records and/or more in-depth surveys with questions focusing more on physical and mental health, symptoms experienced (nausea, pain, etc.), individuals’ experiences trying to become pregnant, and recovery following, seem feasible, and would support deeper exploration of the potential for individualized pregnancy monitoring and analysis. Additionally, the ability of the pregnant participant to view their data over time provides a potential avenue to support behavioral interventions; people may change their behavior, sleep, etc., if they can see that their physiological trajectory is deviating from expectation. With sufficiently diverse and broad participation, it should be possible to learn best practices in steering these trajectories specific to the needs of specific groups or individuals.

Our work supports the notion that remote monitoring is feasible and allows for more fine-grain insight into each individual’s pregnancy between standard clinical appointments. For individuals from lower socioeconomic conditions, and those without easy access to healthcare infrastructure, wearable-supported analyses^18,19,41^ could provide an important supplemental source for monitoring and risk assessment. As recently shown^19^, development of machine learning models from data generated by individuals from diverse backgrounds are feasible. Use of participant-led research may improve the inclusion of individuals who most stand to benefit from wearable device-driven augmentations of standard but more centralized healthcare infrastructure.

In summary, wearable devices make it possible to generate and follow trajectories of physiological variables across the entirety of an individual’s pregnancy journey. Comparison of individual pregnancy journeys to healthy population reference trajectories has now been shown to have relevance from conception through delivery. We found that such comparisons provided indications for early fetal loss as well.

This retrospective analysis was carried out with data volunteered from users of the commercially-available device Oura Ring, supporting the use of wearable devices and community engagement for identifying natural experiments as a complement to controlled experiments or trials. Our findings also support the notion that wearable devices could be used to develop relatively cheap and accessible high-resolution pregnancy monitoring solutions for communities that lack major medical infrastructure. Our work supports the feasibility of working with communities to document their pregnancy journeys, and the use of this approach to develop data-driven, dynamic, personal risk assessment tools.

## Methods

### Wearable device and questionnaire data collection

**Fig. 4:**
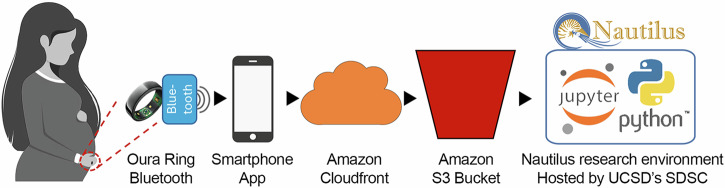
Pathway of data from the individual into analytic cyberinfrastructure. Block diagram showing the flow of data from a pregnant person’s Oura Ring to the UC San Diego Nautilus Portal for analysis in Jupyter notebooks hosted in the Nautilus research environment on the San Diego Supercomputer (SDSC), University of California, San Diego (UCSD). “Jupyter” and “Python” logos are Trademarked and used in accordance with their written use policies. “Nautilus” logo is not protected.

All participants were owners of the Oura Ring Gen2 (Oura Health Oy, Oulu, Finland), a commercial wireless device worn on the finger that contains 3 separate sensors: negative coefficient (NTC) thermistor (resolution of 0.07 °C) to detect distal body temperature (DBT), a tri-axial accelerometer to measure activity (metabolic equivalents, MET, resolution of 60 s), and a photoplethysmography (ppg) sensor (signal sampled at 250 Hz) that measured heart rate (HR), heart rate variability (HRV), and respiratory rate (RR)^7^. All data was wirelessly synced via bluetooth from the ring to the user’s smartphone when the Oura App was in use (Fig. 4). Data were then sent by the app to Oura’s cloud architecture. Oura performed a one-time data push from their secure Amazon storage (S3) to our S3 cloud storage located on San Diego Supercomputer (SDSC) infrastructure.

In addition to the wearable data, participants had the option to answer a questionnaire related to pregnancy via the app. Questionnaire data included approximate age (“What was your approximate age for the majority of this pregnancy? 20–24, 25–29, 30–34, 35–39, 40–44, 45–49), date of conception (“If you know when you likely conceived, please list the most likely date (please leave blank if you don’t have a strong sense of this).”, date the individual knew they were pregnant (‘date know pregnant’, DKP)(“What was the date you first became aware of this pregnancy?”), date of a positive pregnancy test (“If you used a test to confirm this pregnancy, what was your first positive test date?”), date the pregnancy stopped (‘date pregnancy stop’, DPS) (“What was the date this pregnancy concluded?”), and if the pregnancy was considered high risk (“Did a medical professional tell you this was a “high risk” pregnancy? Yes or No”).

### Data

The University of California San Diego (UCSD) Institutional Review Board (IRB, IRB# 201027X) approved all study activities, and all research was performed in accordance with relevant guidelines and regulations and the Declaration of Helsinki. All participants provided informed electronic consent. We did not compensate participants for participation.

For each participant, a single parquet file for nightly aggregated data, also referred to as sleep summary data, contained sleep-related data fields (sleep time start, sleep time end) and the aggregated data fields: temperature max, temperature trend deviation. A single row with the longest sleep duration value for each date was chosen to ensure a single set of sleep measurements per night. High-resolution physiological data included five data modalities stored in separate CSV files for each participant per day. DBT was measured by a negative coefficient (NTC) thermistor (0.07 °C resolution) recorded at 1-minute intervals^7^ 24 hours per day. The Metabolic equivalents activity metrics (MET) was measured by a tri-axial accelerometer and recorded at 1-min intervals 24 h per day. Data measured by a photoplethysmography (ppg) sensor include heart rate (HR) and heart rate variability (HRV) at 5-min intervals and respiratory rate (RR) at 30-s intervals reported only when the wearer is at a rest or sleep state.

### Data processing

564 individuals responded to a survey distributed via the Oura App, and in total, reported 704 unique pregnancies. High-resolution physiological data was generated using the wearable device Oura Ring (Oura Health Oy, Oulu, Finland) and stored in large parquet files on the San Diego Supercomputer Center (SDSC) and accessed via the Nautilus Portal^44^. Data preprocessing included the generation of date-time indexing, normalization of indexes to a ‘local-time’, removal of duplicate time points, filtering of values below the 0.05 quantile and above 0.95 quantiles for each participant, annotation of awake or asleep based on information contained in the sleep summary data, and dropping of Temperature, HR, HRV, and RR values for timepoints where corresponding activity recordings lower than 0.5 MET (resting MET is equal to 1 in these data) to remove potential artifacts from the data caused when a user was not wearing the device.

Further filtering of each modality was applied by dropping values below the 2% quantile and above the 98% quantile calculated for each individual. The following individual daily values aggregated were then calculated for each data type at both 24 h and nightly (8:00 pm to 8:00 am ‘local time’) resolutions: mean, median, average 90–95% quantile (referred to as peak), and average 5–10% quantile (referred to as trough).

### Subjects

Of the pregnancies reported, 52 were those of the respondent’s pregnant partner and 392 took place outside of the wearable device data collection timeframe (September 2019 to February 2022). Of the 224 pregnancies that provided data within this timeframe and also ended in delivery, available data was very sparse (< 40% of days with temperature data in each trimester) for 47 pregnancies for at least one of the trimesters and an additional 76 pregnancies were filtered for reasons such as sparse data directly around pregnancy onset or end of pregnancy and during the time prior to pregnancy. Lastly, 4 preterm pregnancies (<37 weeks gestation) were not included in the analyses, thus in total, 97 full-term pregnancies (>= 37 weeks gestation) were considered due to having daily temperature measurements for at least 40% of days in all three trimesters, and all three trimesters falling between September 2019 and February 2022. Of these, 17 were reported as high risk (Table 4). All full-term pregnancies but two were reported by unique individuals (96 individuals, 97 pregnancies); because the two pregnancies from the same parent occurred in different years, and because they made up only a small percent of the total, we did not attempt to combine them in any way to avoid pseudoreplication, instead including both to maintain full resolution in all pregnancies compared. Within the full-term subset, 5-year age bin counts of participants showed that the majority of pregnancies for this cohort occurred between age 30–39 (Table 4). 34 early fetal loss (EFL) pregnancies were reported and classified by participants as miscarriages, ectopic, chemical pregnancies, terminations, or failure to implant. Failure to implant pregnancies (*n* = 4) were not included in these analyses. Of the 30 remaining EFLs, 3 were filtered due to low first-trimester data completeness and 4 due to another reason such as low prior-to-pregnancy data completeness, resulting in an EFL cohort of 23 pregnancies. In the final EFL cohort, age counts of participants split into 5-year bins were: 25–29 = 1, 30–34 = 5, 35–39 = 14, 40–44 = 3 (Table 4). Of the EFL pregnancies, 7 were reported as high risk. All EFL pregnancies but six were reported by unique individuals (20 individuals, 23 pregnancies); because the lengths of the pregnancies were in different EFL pregnancy length threshold groups and because they were a small percent of the total, we did not attempt to combine them and included them in the final comparisons. No other demographic or risk factors were recorded in the survey.

**Table 4:** Cohort Demographics and pregnancy outcomes by maternal age

	Full-term		Early fetal loss	
Age group	All	High Risk	All	High Risk
25–29	13	2	1	0
30–34	36	2	5	0
35–39	29	4	14	5
40–44	17	7	3	2
45–49	2	2	0	0
Total count	97	17	23	7

All available participant demographics for each unique pregnancy included age and patient-reported high-risk labels during the 97 full-term and 23 Early Fetal Loss pregnancies for 96 and 20 participants, respectively.

### Analysis methods

#### Alignment of pregnancies

Alignment of (full-term or EFL) pregnancies was performed using two dates responders provided in the survey: date the individual knew they were pregnant (referred to as ‘date know pregnant’ (DKP) and often the same date as the date of their first positive pregnancy test) or reported date that the pregnancy stopped (‘date the pregnancy stopped’, DPS). The date the individual knew they were pregnant aligned well with 28 days after the most recent trough of nightly maximum measurement, which has been previously characterized as the start of menstruation^45^, thus alignment by that date was used to align near the start of pregnancy.

#### Box and Whisker plots of full-term pregnancies by trimester

Median, lower quartile, and upper quartile of the trimester means for the 97 full-term pregnancies were plotted for five periods: 13 weeks before pregnancy onset (day -91 to date know pregnant (day 0) - 28 days, 13–14 weeks for each trimester (trimester 1: date know pregnant (day 0) - 28 days to day 63 [91 days], trimester 2: day 64 to day 154 [91 days], trimester 3: day 155 to day 252 [97 days]) and the 13 weeks following pregnancy (trimester 4: day 252 to day 343, [91 days]). Statistical significance between trimester means was calculated using the Mann-Whitney-Wilcoxon two-sided test with Bonferroni Correction for the 4 comparisons using the add_stat_annotation function from the *statannot* (version 0.2.3, https://pypi.org/project/statannotations/) python (version 3.11.5) package.

#### Median and quantile analysis of full-term pregnancies

For each data type, all pregnancies were aligned by ‘date know pregnant’ and the median and quantile ranges were calculated across the cohort for each day using built-in *pandas* (version 2.1.0)^46^ functions.

#### Z-score, median, and confidence interval analysis

For each individual and each data type, daily aggregated data was z-scored by calculating the mean and standard deviation of data preceding pregnancy onset (DKP- 60 days to DKP - 30 days) then calculating the z-score value for all dates (z = (*x* - *μ*_-60-30_)/(*σ*_-60-30_)). After alignment of pregnancies either by DKP or DPS, a rolling 7-day mean was calculated, then the median value and 95% CI was calculated for each day (*scipy*^47^ package; version 1.10.1, https://scipy.org/).

#### Cumulative multimodal distance

For each participant, for each day, we generated z-scored values for the 4 most complete data types (temp peak, temp trough, HR, HRV) and stored the daily means as 4-dimensional points. To calculate a multimodal distance for the >35 year-old subgroup from the <35 year-old subgroup, we calculated the mean for each data type for each day using daily values from the <35 subgroup. Then, we computed the Euclidean distance between each >35 participant and the <35 group mean at each time point. To calculate an individual’s multimodal distances for <35 population, we computed the Euclidean distance between each participant <35 and the mean of all other <35 participants at each timepoint (leave-one-out method). Each individual’s cumulative distance was generated as the daily sum of all preceding daily distance values for that participant. The cumulative values at 40 weeks for each subgroup were compared using a Kruskal-Wallis test using *scipy*^47^ python package (version 1.10.1, https://scipy.org/).

#### Generalized estimating equation statistical analysis

Data was preprocessed by performing linear interpolation and applying a rolling 3-day mean. Z-scoring was performed by calculating the mean and standard deviation of data preceding pregnancy onset (DKP- 60 days to DKP - 30 days) for all but one of the 23 EFL pregnancies as data before onset was not available (22 EFLs total). Early fetal loss (EFL) pregnancies were separated into groups based on pregnancy length from ‘date know pregnant’ to ‘date pregnancy stop’ in days (thresholds: 14, 28, 40, and 60). For each EFL within each length group, the 7 days following ‘date pregnancy stop’ were stored along with the same days from 5 randomly selected full-term pregnancies (e.g. if an EFL occurred on day 10, values were selected from days 10 through 16 for that pregnancy and from 5 other, full-term pregnancies to ensure time-matched analysis for each individual’s EFL date); in this way, if there are 5 EFLs in a length group, data from 25 full-term pregnancies was also stored in the same array. The Generalized Estimating Equation (GEE) analysis was performed using the python package statsmodels^48^ (version 0.14.0, https://www.statsmodels.org/). The model used z-scored temperature as the dependent variable (dv), with the day following the EFL, the pregnancy category (full-term or early fetal loss), and EFL pregnancy threshold as the independent variables (iv).

## Data Availability

Oura’s data use policy does not permit us to make wearable device data (collected via the Oura Ring) available to third parties. Those seeking to reproduce findings in this manuscript should contact the corresponding author B.L.S.

## Code availability

Code will be made available through the UC San Diego Library Digital Collections repository with the DOI generated upon publication.

## References

[1] Guidelines for perinatal care. (American Academy of Pediatrics; The American College of Obstetricians and Gynecologists, 2017).

[2] Hunter, S. & Robson, S. C. Adaptation of the maternal heart in pregnancy. *Heart* **68**, 540–543 (1992). 10.1136/hrt.68.12.540 PMC1025680 1467047

[3] Kuo, C. D., Chen, G. Y., Yang, M. J., Lo, H. M. & Tsai, Y. S. Biphasic changes in autonomic nervous activity during pregnancy. *Br. J. Anaesth.* **84**, 323–329 (2000). 10793590 10.1093/oxfordjournals.bja.a013433

[4] Green, L. J. et al. Gestation-specific vital sign reference ranges in pregnancy. *Obstet. Gynecol.* **135**, 653–664 (2020). 32028507 10.1097/AOG.0000000000003721

[5] Jimah, T. et al. A micro-level analysis of physiological responses to COVID-19: continuous monitoring of pregnant women in California. *Front. Public Health* **10**, 808763 (2022). 35462830 10.3389/fpubh.2022.808763 PMC9021503

[6] Niela-Vilén, H. et al. Pregnant women’s daily patterns of well-being before and during the COVID-19 pandemic in Finland: Longitudinal monitoring through smartwatch technology. *PLoS ONE* **16**, e0246494 (2021). 33534854 10.1371/journal.pone.0246494 PMC7857616

[7] Mason, A. E. et al. Detection of COVID-19 using multimodal data from a wearable device: results from the first TemPredict Study. *Sci. Rep.* **12**, 3463 (2022). 35236896 10.1038/s41598-022-07314-0 PMC8891385

[8] Radin, J. M., Wineinger, N. E., Topol, E. J. & Steinhubl, S. R. Harnessing wearable device data to improve state-level real-time surveillance of influenza-like illness in the USA: a population-based study. *Lancet Digital Health* **2**, e85–e93 (2020). 33334565 10.1016/S2589-7500(19)30222-5 PMC8048388

[9] Temple, D. S. et al. Wearable sensor-based detection of influenza in presymptomatic and asymptomatic individuals. *J. Infect. Dis.* **227**, 864–872 (2023). 35759279 10.1093/infdis/jiac262 PMC9384446

[10] Domingo-Lopez, D. A. et al. Medical devices, smart drug delivery, wearables and technology for the treatment of diabetes mellitus. *Adv. Drug Deliv. Rev.* **185**, 114280 (2022). 35405298 10.1016/j.addr.2022.114280

[11] Perry, A. S. et al. Association of longitudinal activity measures and diabetes risk: an analysis from the National Institutes of health all of us research program. *J. Clin. Endocrinol. Metab.* **108**, 1101–1109 (2023). 36458881 10.1210/clinem/dgac695 PMC10306083

[12] Wang, Y.-C. et al. Current advancement in diagnosing atrial fibrillation by utilizing wearable devices and artificial intelligence: a review study. *Diagnostics* **12**, 689 (2022). 35328243 10.3390/diagnostics12030689 PMC8947563

[13] Lubitz, S. A. et al. Detection of atrial fibrillation in a large population using wearable devices: the fitbit heart study. *Circulation* **146**, 1415–1424 (2022). 36148649 10.1161/CIRCULATIONAHA.122.060291 PMC9640290

[14] Penders, J., Altini, M., Van Hoof, C. & Dy, E. Wearable sensors for healthier pregnancies. *Proc. IEEE* **103**, 179–191 (2015). 10.1109/JPROC.2014.2387017

[15] Walter, J. R., Xu, S., Stringer, J. S. & Rogers, J. A. The Future of Remote Monitoring for Pregnancy. (2022). PMC10727511 38111590

[16] Maugeri, A., Barchitta, M. & Agodi, A. How wearable sensors can support the research on foetal and pregnancy outcomes: a scoping review. *JPM* **13**, 218 (2023). 36836452 10.3390/jpm13020218 PMC9961108

[17] Alim, A. & Imtiaz, M. H. Wearable sensors for the monitoring of maternal health—a systematic review. *Sensors* **23**, 2411 (2023). 36904615 10.3390/s23052411 PMC10007071

[18] Erickson, E. N. et al. Predicting labor onset relative to the estimated date of delivery using smart ring physiological data. *npj Digit. Med.* **6**, 153 (2023). 37598232 10.1038/s41746-023-00902-y PMC10439919

[19] Ravindra, N. G. et al. Deep representation learning identifies associations between physical activity and sleep patterns during pregnancy and prematurity. *npj Digit. Med.* **6**, 171 (2023). 37770643 10.1038/s41746-023-00911-x PMC10539360

[20] Ryu, D. et al. Comprehensive pregnancy monitoring with a network of wireless, soft, and flexible sensors in high- and low-resource health settings. *Proc. Natl Acad. Sci. USA* **118**, e2100466118 (2021). 33972445 10.1073/pnas.2100466118 PMC8157941

[21] Kominiarek, M. A., Balmert, L. C., Tolo, H., Grobman, W. & Simon, M. A feasibility study of activity tracking devices in pregnancy. *BMC Pregnancy Childbirth* **19**, 401 (2019). 31684889 10.1186/s12884-019-2557-3 PMC6829855

[22] Sarhaddi, F. et al. Long-term IoT-based maternal monitoring: system design and evaluation. *Sensors* **21**, 2281 (2021). 33805217 10.3390/s21072281 PMC8036648

[23] Nulty, A. K., Chen, E. & Thompson, A. L. The Ava bracelet for collection of fertility and pregnancy data in free-living conditions: an exploratory validity and acceptability study. *DIGITAL HEALTH* **8**, 205520762210844 (2022). 10.1177/20552076221084461 PMC8918962 35295766

[24] Runkle, J. et al. of wearable sensors for pregnancy health and environmental monitoring: descriptive findings from the perspective of patients and providers. *Digital Health* **5**, 205520761982822 (2019). 10.1177/2055207619828220 PMC6376550 30792878

[25] Wakefield, C., Yao, L., Self, S. & Frasch, M. G. Wearable technology for health monitoring during pregnancy: an observational cross-sectional survey study. *Arch. Gynecol. Obstet.* **308**, 73–78 (2022). 35831759 10.1007/s00404-022-06705-y PMC9281287

[26] Lopez, B. D. B., Aguirre, J. A. A., Coronado, D. A. R. & Gonzalez, P. A. Wearable technology model to control and monitor hypertension during pregnancy. in 2018 13th Iberian Conference on Information Systems and Technologies (CISTI) 1–6 (IEEE, 2018). 10.23919/CISTI.2018.8399200.

[27] Souza, R. T. et al. Identification of earlier predictors of pregnancy complications through wearable technologies in a Brazilian multicentre cohort: Maternal Actigraphy Exploratory Study I (MAES-I) study protocol. *BMJ Open* **9**, e023101 (2019). 31005906 10.1136/bmjopen-2018-023101 PMC6500316

[28] Polsky, S. & Garcetti, R. CGM, Pregnancy, and Remote Monitoring. Diabetes Technology & Therapeutics 19, S-49-S-59 (2017). 10.1089/dia.2017.0023 PMC5467097 28585876

[29] Gupta, Y. et al. Continuous glucose monitoring system profile of women stratified using different levels of glycated hemoglobin (HbA1c) in early pregnancy: a cross-sectional study. *Adv. Ther.* **40**, 951–960 (2023). 36550320 10.1007/s12325-022-02405-w

[30] Márquez-Pardo, R. et al. Continuous glucose monitoring and glycemic patterns in pregnant women with gestational Diabetes Mellitus. *Diabetes Technol. Therapeutics* **22**, 271–277 (2020). 10.1089/dia.2019.0319 31638416

[31] Polsky, S. et al. Continuous glucose monitor use with and without remote monitoring in pregnant women with type 1 diabetes: a pilot study. *PLoS ONE* **15**, e0230476 (2020). 32298269 10.1371/journal.pone.0230476 PMC7162510

[32] Jimah, T. et al. A technology-based pregnancy health and wellness intervention (Two Happy Hearts): case study. *JMIR Form. Res.* **5**, e30991 (2021). 34787576 10.2196/30991 PMC8663690

[33] Ng, A. et al. Predicting the next-day perceived and physiological stress of pregnant women by using machine learning and explainability: algorithm development and validation. *JMIR Mhealth Uhealth* **10**, e33850 (2022). 35917157 10.2196/33850 PMC9382551

[34] Saarikko, J. et al. Continuous 7-month internet of things–based monitoring of health parameters of pregnant and postpartum women: prospective observational feasibility study. *JMIR Form. Res.* **4**, e12417 (2020). 32706696 10.2196/12417 PMC7414406

[35] Sarhaddi, F. et al. Trends in heart rate and heart rate variability during pregnancy and the 3-month postpartum period: continuous monitoring in a free-living context. *JMIR Mhealth Uhealth* **10**, e33458 (2022). 35657667 10.2196/33458 PMC9206203

[36] Rowan, S. P., Lilly, C. L., Claydon, E. A., Wallace, J. & Merryman, K. Monitoring one heart to help two: heart rate variability and resting heart rate using wearable technology in active women across the perinatal period. *BMC Pregnancy Childbirth* **22**, 887 (2022). 36451120 10.1186/s12884-022-05183-z PMC9710029

[37] Smarr, B. L. et al. Feasibility of continuous fever monitoring using wearable devices. *Sci. Rep.* **10**, 21640 (2020). 33318528 10.1038/s41598-020-78355-6 PMC7736301

[38] Wilcox, A. J. et al. Incidence of early loss of pregnancy. *N. Engl. J. Med.* **319**, 189–194 (1988). 3393170 10.1056/NEJM198807283190401

[39] Chard, T. 11 Frequency of implantation and early pregnancy loss in natural cycles. *Baillière’s Clin. Obstet. Gynaecol.* **5**, 179–189 (1991). 1855339 10.1016/S0950-3552(05)80077-X

[40] Regan, L. & Rai, R. Epidemiology and the medical causes of miscarriage. *Best. Pract. Res. Clin. Obstet. Gynaecol.* **14**, 839–854 (2000). 10.1053/beog.2000.0123 11023804

[41] Grant, A. & Smarr, B. Feasibility of continuous distal body temperature for passive, early pregnancy detection. *PLOS Digit Health* **1**, e0000034 (2022). 36812529 10.1371/journal.pdig.0000034 PMC9931282

[42] Jolly, M. The risks associated with pregnancy in women aged 35 years or older. *Hum. Reprod.* **15**, 2433–2437 (2000). 11056148 10.1093/humrep/15.11.2433

[43] Kenny, L. C. et al. Advanced maternal age and adverse pregnancy outcome: evidence from a large contemporary cohort. *PLoS ONE* **8**, e56583 (2013). 23437176 10.1371/journal.pone.0056583 PMC3577849

[44] Purawat, S. et al. TemPredict: A Big Data Analytical Platform for Scalable Exploration and Monitoring of Personalized Multimodal Data for COVID-19. in 2021 IEEE International Conference on Big Data (Big Data) 4411–4420 (IEEE, 2021). 10.1109/BigData52589.2021.9671441.

[45] Chen, W., Kitazawa, M. & Togawa, T. Estimation of the biphasic property in a female’s menstrual cycle from cutaneous temperature measured during sleep. *Ann. Biomed. Eng.* **37**, 1827–1838 (2009). 19551509 10.1007/s10439-009-9746-6

[46] McKinney, W. Data Structures for Statistical Computing in Python. in 56–61 (2010). 10.25080/Majora-92bf1922-00a.

[47] Virtanen, P. et al. SciPy 1.0: fundamental algorithms for scientific computing in Python. *Nat. Methods* **17**, 261–272 (2020). 32015543 10.1038/s41592-019-0686-2 PMC7056644

[48] Seabold, S., Perktold, J. Statsmodels: Econometric and statistical modeling with python. Proceedings of the 9th Python in Science Conference. 2010.

